# Appropriate First-Line Regimens to Combat *Helicobacter pylori* Antibiotic Resistance: An Asian Perspective

**DOI:** 10.3390/molecules20046068

**Published:** 2015-04-08

**Authors:** Muhammad Miftahussurur, Yoshio Yamaoka

**Affiliations:** 1 Department of Environmental and Preventive Medicine, Oita University Faculty of Medicine, Yufu 879-5593, Japan; E-Mail: miphto@oita-u.ac.jp; 2 Gastroentero-Hepatology Division, Department of Internal Medicine, Airlangga University Faculty of Medicine, Surabaya 60131, Indonesia; 3 Institute of Tropical Disease, Airlangga University, Surabaya 60115, Indonesia; 4 Department of Gastroenterology and Hepatology, Baylor College of Medicine and Michael DeBakey Veterans Affairs Medical Center, Houston, TX 77030, USA

**Keywords:** *Helicobacter pylori*, Asia, antibiotic resistance, clarithromycin (CAM), amoxicillin (AMX), metronidazole (MNZ), proton pump inhibitor (PPI), CYP2C19 polymorphisms

## Abstract

Asia has the largest population of any continent and the highest incidence of gastric cancer in the world, making it very important in the context of *Helicobacter pylori* infection. According to current guidelines, standard triple therapy containing a proton pump inhibitor (PPI) and two antibiotics; amoxicillin (AMX) and clarithromycin (CAM) or metronidazole (MNZ), is still the preferred first-line regimen for treatment of *H. pylori* infection. However, the efficacy of legacy triple regimens has been seriously challenged, and they are gradually becoming ineffective. Moreover, some regions in Asia show patterns of emerging antimicrobial resistance. More effective regimens including the bismuth and non-bismuth quadruple, sequential, and dual-concomitant (hybrid) regimens are now replacing standard triple therapies as empirical first-line treatments on the basis of the understanding of the local prevalence of *H. pylori* antimicrobial resistance. Selection of PPI metabolized by the non-enzymatic pathway or minimal first pass metabolism and/or increasing dose of PPI are important to increase *H. pylori* eradication rates. Therefore, local antibiotic resistance surveillance updates, selection of appropriate first-line regimens with non-enzymatic PPI and/or increased doses of PPI, and detailed evaluation of patients’ prior antibiotic usage are all essential information to combat *H. pylori* antibiotic resistance in Asia.

## 1. Introduction

*Helicobacter pylori* infection is accepted as the primary cause of chronic gastritis [1]. Moreover, severe atrophic gastritis accompanying intestinal metaplasia caused by persistent *H. pylori* infection is closely related to the development of gastric cancer (GC) [2]. Asia is a very important continent in the context of *H. pylori* infection. It has the largest population of any continent (4.4 billion people) and the highest incidence of GC in the world, with an age-standardized incidence rate (ASR) of 15.8/100,000 (available from the International Agency for Research on Cancer; GLOBOCAN2012, http://globocan.iarc.fr/). The population of India is approximately 1.2 billion people; if *H. pylori* prevalence was 60%, then more than 726 million individuals in India would be infected with *H. pylori*. Furthermore, the estimated prevalence of duodenal ulcer in India is 3 per cent, meaning that at least 18 million people could need anti-*H. pylori* therapy (approximately 50,000 per day if treated over one year) [3].

**Figure 1:**
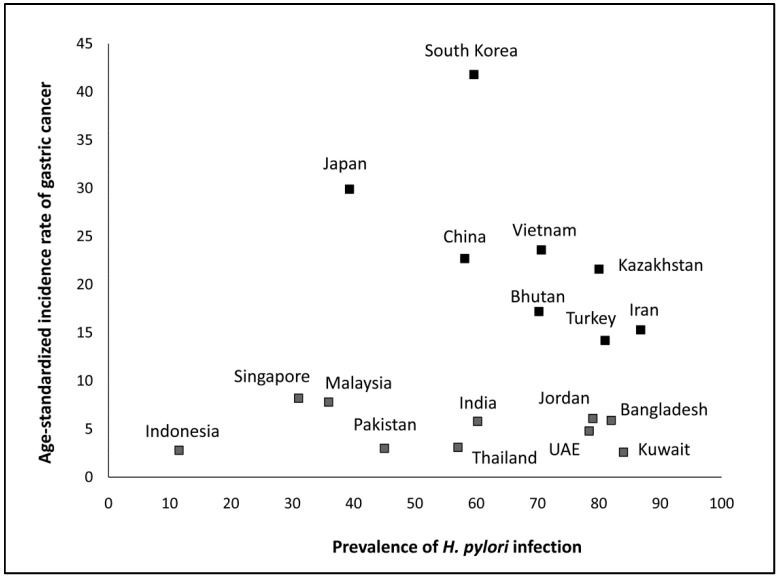
The association of *H. pylori* infection rate with ASR for GC from 18 countries and four regions in Asia. Although the incidence rate of GC in several regions in Asia tends to mirror the prevalence rate of *H. pylori* infection, several countries were categorized as an “Asian enigma”; India, Pakistan, Bangladesh, Jordan, United Arab Emirates and Kuwait. Interestingly, Indonesia has a low prevalence rate of *H. pylori* infection and categorized as low-risk country.

In Asia, there is a considerable geographic variation in the prevalence of *H. pylori* infection. The incidence rate of GC in several regions in Asia tends to mirror the prevalence of *H. pylori* infection [4]. Figure 1 summarizes the association of *H. pylori* infection rates with ASR for GC from 18 countries and four regions in Asia. East Asian countries (Korea, China, and Japan) [5,6,7], Vietnam in Southeast Asia [8] and Kazakhstan in Southern Central Asia [9] with the high prevalence rates of *H. pylori* infection were categorized as the high-risk areas for GC (ASR >20/100 000), whilst two Western Asian countries (Turkey and Iran) [10,11], and Bhutan in Southeast Asia [12] with high prevalence rates of *H. pylori* infection were categorized as intermediate-risk countries (ASR 11–20/100,000). However, a high prevalence of *H. pylori* infection is not always associated with a high incidence of GC. For example, despite the high infection rate in South Asian countries (India, Pakistan, and Bangladesh) [13] and three Western Asian countries (Jordan, United Arab Emirates, and Kuwait) [10,14,15], the incidence of GC in those regions is low (ASR <10/100,000), which is known as an “Asian enigma” [13]. Interestingly, Indonesia has a low prevalence rate of *H. pylori* infection and is categorized as a low-risk country. Using five different methods, the prevalence rate of *H. pylori* in Indonesia was measured as only 11.5% [16].

The emergence of drug resistance in *H. pylori* eradication is a serious problem. Several indications and regimens were recommended by guidelines from several regions including Asia Pacific, China, Japan, and South Korea. However, in the following years, the legacy efficacy some regimens has been seriously challenged, and they are becoming ineffective. Moreover, some regions in Asia show patterns of emerging antimicrobial resistance. In Asian countries with a high prevalence of the infection; with increased resistance to the antibiotics used to treat it, which might increase the recurrence rate of the infection; and with high morbidity and mortality rates caused by *H. pylori* infection-associated pathologies, prevention should be the ultimate solution, and vaccines have been suggested as a cost-effective alternative to slow down the emergence of drug resistance by decreasing the infection rate and hence antibiotic usage [17]. However to date an efficient vaccine has not yet been developed. The complicated host immune response, along with considerable genetic diversity and due to a long period between *H. pylori* acquisition and manifestation of disease, especially GC, hold up vaccine development [17].

Several possible factors causing progressive eradication therapy failure include antibiotic resistance, poor compliance, high gastric acidity, high bacterial load, and cytochrome P450 2C19 (CYP2C19) polymorphism [18]. Local antibiotic resistance screening updates, selection of appropriate first-line regimens based on antibiotic resistance surveillance, followed by detailed evaluation of patient prior antibiotic usage, are the main factors to prevent repeated treatment courses resulting in both multiple side effects (and therefore poor patient adherence and quality of life) and spreading of secondary antibiotic resistance [19].

In this review we summarize the current antimicrobial resistance rate in Asia, analyze and compare the most important results of various established eradication protocols, suggest first line regimens, and the importance of the proton pump inhibitor (PPI) as a component of *H. pylori* eradication regimens to influence overall therapy success in the Asian population.

## 2. *H. pylori* Antibiotic Resistance Rates in Asia

The prevalence of antibiotic resistance is now increasing worldwide and varies by the geographic area; it is generally higher in developing countries than in developed regions [20]. In addition, the antibiotic resistance rate often parallels the antibiotic consumption rates in the population [21,22]. Table 1 summarizes antibiotic resistance rates from 16 countries and four regions in Asia. Clarithromycin (CAM) resistance has been shown to be associated with any one of three well-known point mutations in the 23S rRNA gene of *H. pylori*; these three mutations are responsible for more than 90% of CAM resistance cases in developed countries [23]. Interestingly, the point mutations inducing CAM resistance in Asian countries differ from those in Europe and North America [24]. Additional mutations such as T2183C and A2223G have been frequently found to be the cause of observed CAM resistance, while the A2143G mutation, which has a much stronger impact than A2142G, and A2142C [25], which are responsible for 90% of cases of primary CAM resistance in *H. pylori* strains isolated in Western countries [26], accounted only for 23% of resistant strains in Asia [26]. In East Asian countries, high levels of CAM resistance have been recorded. For example, in Japan the annual surveillance for 5 years conducted between 2002 and 2006 showed that the mean nationwide CAM resistance rates had increased from 18.9% in 2002 to 27.2% in 2006 [27]. Increasing rates of CAM primary resistance have also been reported in South Korea (17.2%–23.7%) and China (14.8% to 65.4%) [28,29]. In South Asia, India has a higher resistance rate than Pakistan [30,31]. In Western Asia, resistance rates have been increasing over the last 20 years. In Iran, CAM resistance has increased from 1.4% in 1997 to 26.5% in 2013 [32,33]. Turkey and Bahrain also have high rates of CAM resistance [34,35]. Interestingly, whilst resistance rates in Vietnam and Indonesia are considered to be high [36,37], the resistance rates are very low in Thailand and Singapore (3.7% and 6%, respectively) [38,39]. Moreover, in Bhutan and Malaysia, no *H. pylori* strains showed resistance to CAM [40,41]. This suggested that Southeast Asia is the region of Asia with the lowest CAM resistance rates.

Metronidazole (MNZ) is another agent frequently included in regimens to eradicate *H. pylori*. Therefore, the presence of MNZ resistance may also affect therapeutic outcomes. The mechanisms of MNZ resistance are complex, but are largely associated with inactivating mutations of the *rdxA* and *frxA* genes, which encode reductases required for the activation of MNZ [42]. However, development of MNZ resistance can occur independently of these mutations, suggesting alternative, as yet unknown, resistance mechanisms exist [43]. In East Asia, China has the highest prevalence of MNZ resistance (56.6%–95.4%) [29,44]. Prevalence of resistance in Hong Kong and South Korea are considered to be high [45,46,47]. However, contrary to the general phenomenon whereby prevalence of CAM resistance tends to be much lower than that of MNZ, the MNZ resistance rate in Japan is only 3.3%–4.9%, as recorded by annual surveillance for 5 years [27]. In Southeast Asia, only Thailand and Malaysia [38,48] have MNZ resistance rates below 40%. Rates of resistance to MNZ were found to be high in Singapore and Vietnam [39,49]. Bhutan (82.9%) and Indonesia (100%) have the highest prevalence of MNZ resistance in this region [37,40]. High prevalence of MNZ resistance was also reported in Western and Southern Asia [30,31,34,35,50,51].

**Table 1:** Antibiotic resistance rates from 16 countries and four regions in Asia.

Ref	Country	City	Year	Patients	Methods	CAM	MNZ	LVX	TCN	AMX	Others
**East Asia**											
[27]	Japan	Multicentre	2002–2003	1069	ADM	18.9%	4.9%	-	-	15.2	-
			2003–2004	1381	ADM	21.1%	5.3%	-	-	21.4%	-
			2004–2005	1257	ADM	27.7%	3.3%	-	-	16.3%	
[52]	Japan	Tokyo	1996–2008	3521	DDM	16.4%	-	-	-	0.03%	minocycline (0.06%)
[29]	China	Beijing	2000–2009	290	E-test	23.8%	56.6%	36.9%	1.0%	0.3%	MOX (41.2%)
[44]	Southeast China	2 provinces	2010–2012	17,731	ADM	21.5%	95.4%	20.6%	-	0.1%	furazolidone (0.1%), gentamicin (0.1%)
[47]	China	Hongkong	NM	83	ADM	10.8%	49.4%	-	-	-	
[53]	Taiwan	Taichung	1998–2004	218	E-test	8.3%	31.7%	-	-	0.0%	
[54]	Taiwan	Hualien	2004–2005	133	E-test	13.5%	51.9%	-	0.0%	0.0%	
[28]	South Korea	Seoul	2003–2005	70	ADM	22.9%	34.3%	5.7%	18.6%	7.1%	AZT (25.7%), MOX (5.7%)
			2006–2008	201	ADM	25.5%	26.0%	27.4%	32.8%	9.5%	AZT (27.4%), MOX (27.9%)
			2009–2012	162	ADM	37.0%	35.8%	34.6%	35.2%	18.5%	AZT (34.0%), MOX (34.6%)
[46]	South Korea	Seoul	2004–2005	65	ADM	13.8%	66.2%	21.5%	12.3%	18.5%	AZT (32.3%), CIP (33.8%), MOX (21.5%)
**West Asia**											
[50]	Iran	Sari	2009	197	DDM	45.2%	65.5%	37.1%	-	23.9%	CIP (34.5%), furazolidone (61.4%)
[55]	Iran	Shiraz	2008–2009	121	E-test	4.9%	43.8%	-	3.3%	15.7%	
[34]	Turkey	Elazig	2009–2010	61	DDM	21.3%	42.6%	3.3%	0.0%	0.0%	
[51]	Saudi Arabia	Jeddah	2002	223	DDM	4.0%	80.0%	-	0.4%	1.3%	
[35]	Bahrain	Bahrain	1998–1999	83	E-test	32.5%	57.0%	-	0.0%	0.0%	
**South Asia**											
[31]	India	Gujarat	2008–2011	80	DDM	58.8%	83.8%	72.5%	53.8%	72.5%	CIP (50%)
[56]	India	Multicenter	NM	259	E-test	44.7%	77.9%	-	-	32.8%	
[30]	Pakistan	Karachi	2005–2008	178	NM	36.0%	89.0%	-	12.0%	37.0%	ofloxacin (18.5%)
**South East Asia**											
[37]	Indonesia	Jakarta	2006	72	DDM	27.8%	100.0%	1.4%	-	19.4%	CIP (6.9%), MOX (1.4%), OFX (6.9%)
[38]	Thailand	Nationwide	2004–2012	400	E-test	3.7%	36.0%	7.2%	1.7%	5.2%	CIP (7.7%)
[39]	Singapore	Singapore	1995–1998	282	DDM	6.0%	46.0%	-	-	-	
[48]	Malaysia	Selangor	2004–2007	187	E-test	2.1%	36.4%	1.0%	0.0%	0.0%	CIP (0.0%)
[40]	Buthan	3 cities	2010	111	E-test	0.0%	82.9%	2.7%	0.0%	0.0%	CIP (2.7%)
[49]	Vietnam	2 cities	2008	103	E-test	33.0%	69.9%	18.4%	5.8%	0.0%	

Abbreviations: ADM: Agar Dilution Method, DDM: Disk diffusion method, E-test: Epsilometer test, NM: Not mentioned, CAM: clarithomycin, MNZ: metronidazole, LVX : levofloxacin, MOX: moxifloxacin, AMX: amoxicillin, CIP: ciprofloxacin, TCN: tetracycline, AZT: azithromycin, OFX: ofloxacin.

Loss of penicillin-binding protein is known to be associated with amoxicillin (AMX) resistance [25]. However, research for the rates of AMX resistance is limited. Although most studies estimates rates of resistance to AMX as <1% in China, Turkey, Bahrain, Malaysia, Bhutan and Vietnam, the resistance rate in Japan is >10% [27]. Increasing AMX primary resistance rates have also been reported in South Korea (7.1%–18.5%) [28]. India and Pakistan also have high resistance rates to AMX (72.5% and 37.0%, respectively) [30,31]. In Southeast and Western Asia, only Indonesia and Iran have reported to have high resistance rates (19.4% and 23.9%, respectively) [37,50].

Fluoroquinolones, especially levofloxacin (LVX)-based triple therapy, achieve good *H. pylori* cure rates. As with other bacteria, resistance of *H. pylori* to fluoroquinolones is due to point mutations in the quinolone resistance determining regions of *gyrA*. Rates of resistance to fluoroquinolones also mirror the level of use of these kinds of drugs [21]. In Asia, fluoroquinolone resistance rates differ among countries. In Taiwan the resistance rate is low (8.8%), but the resistance is higher in the Southeast coastal region of China and Beijing, China. A high rate of primary moxifloxacin resistance was also reported in Beijing [29,57]. Moreover, the primary LVX and moxifloxacin resistance rate in South Korea rose from 5.7% in 2003–2005 to 34.6% in 2009–2012 [28]. In Western and Southern Asia, Turkey was found to have a low LVX resistance rate [34], whereas high LVX and ciprofloxacin (CIP) resistance rates were reported in Iran and India (72.5% and 50.0%, respectively) [31,50]. Although resistance rates of 18.4% have been reported in Vietnam [36], LVX resistance rates in Southeast Asia are otherwise low [12,37,38,48].

The mechanisms of tetracycline (TCN) resistance has been characterized as a change in three contiguous nucleotides in the 16S rRNA gene (AGA 926-928RTTC). Resistance to TCN is very low, or even absent, in most Asian countries [21]. Indeed, the resistance to TCN has been shown to be infrequent in Beijing, occurring in 1 of 49 (2.0%) cases in 2006–2007, 0 of 63 cases in 2008, and 1 of 52 (1.9%) cases in 2009 [29]. Resistance rates in Saudi Arabia, Thailand, and Vietnam have been reported to be low, and absent in Taiwan, Turkey, Bahrain, Malaysia, and Bhutan [34,35,40,48]. In contrast, higher values were found in South Korea: these increased from 18.6% in 2003–2005 to 35.2% in 2009–2012 [28]. TCN resistance rates are also high in South Asia [38,49,51].

Unfortunately, data for antibiotics resistance in some studies were inconsistent, even within the same country. For examples, in Japan, contrary with nationwide survey results [27], CAM resistance rates on a study in Tokyo were below 20%. The similar problem was also found in South Korea, Iran and India. The differences could be explained by several reasons. First, although studies were conducted in the same area, *H. pylori* strains were isolated from different hospitals and periods. In addition, some regions in Asia still have still only old local antimicrobial resistance data, from more than 10 years ago. This represents a limitation for comparison between different countries/areas. Second, there are several problems with antimicrobial susceptibility testing of *H. pylori* due to a lack of standard testing and interpretative criteria for susceptibility recommendations [58,59]. The disk-diffusion test is simple, cheap and easy, therefore this method has been frequently used [60]. In contrast, agar or broth dilution methods are difficult to perform routinely [61]. The E-test has been recommended for *H. pylori* because it has a more stable pattern to antibiotic release and tolerance with a prolonged incubation [62]. On the other hand, only the official breakpoint for CAM resistance has been designated for *H. pylori* isolates by the NCCLS, with a MIC breakpoint of CAM at >1.0 µg/mL. Therefore, standard acceptable limits for other antibiotics varied among studies.

## 3. The Various Established Eradication Regimens in Asia

Guidelines for the management of *H. pylori* infection are still evolving, and vary according to the geographic area. First-line, alternative first-line, second-line, or even third-line therapies have been proposed. Recent guidelines proposed for the Asia-Pacific region, World Gastroenterology Organization global guidelines for developing countries, and guidelines for three countries in East Asia are summarized in Table 2 [63,64,65,66]. However, some regimens were confined to very small geographic districts, and therefore were not the therapeutic guidelines that might be valid worldwide.

According to current guidelines, standard triple therapy containing a PPI and two antibiotics, AMX and CAM or MNZ, is still the first-line regimen for treatment of *H. pylori* infection [63,64,65,66]. However, in recent years, the efficacy of legacy triple regimens has been seriously challenged and cure rates lower than 70% are now reported in many countries [19]. In East Asia, the revised 2013 version of the Japanese guideline recommends a lower dose of antibiotics for a shorter duration (7 days) than guidelines from China or South Korea. No 14-day treatments or bismuth-based regimens are recommended as first- or second-line treatments in Japan. The first-line therapy approved by the Japanese health insurance system is CAM-containing triple therapy. Therefore, many Japanese physicians currently prescribe CAM-containing triple therapy according to the national health insurance system, even with the knowledge that this regimen is not effective in areas with a high prevalence of CAM-resistant strains. Although the Japanese health insurance system has not approved an MNZ-containing regimen as a first-line eradication regimen yet, it would be a better future first-line therapy in Japan than CAM-containing triple therapy. When *H. pylori* eradication fails in patients undergoing CAM-based triple therapy, MNZ-based triple therapy can be used as a second-line eradication regimen using the national health insurance system in Japan. This second-line therapy was reported to be highly successful, with a cure rate of more than 90% [67,68]. Using the MNZ breakpoint of 8 µg/mL established by the European Study Group, resistance rates did not change from 2002–2003 and 2004–2005 (4.9% and 3.3%, respectively) [27].

Recent meta-analysis from 104 studies in South Korea comprising 42,124 patients found the overall cure rate for triple therapy was 74.6% (95% CI 72.1%–77.2%) by intention-to-treat (ITT) and 82.0% (95% CI, 80.8%–83.2%) by per-protocol (PP), and the cure rate was decreased significantly from 1998 to 2013 [69]. Several studies in West and South Asia also showed the ineffectiveness of triple therapy as a first line treatment [32,70]. Moreover, substitution with MNZ in Vietnam still showed similar results with a cure rate <70% [71]. In Asia, only Japan, Thailand and Malaysia have populations with <40% MNZ resistance (Table 1), and the Maastricht III Consensus Report stated that use of the CAM is preferable for these countries. In contrast, a regimen including MNZ is not suitable and should not be chosen as first-line treatment therapy in most other Asian countries. Alternatively, PPI + CAM + AMX treatment is recommended as first choice treatment in populations with less than 15%–20% CAM resistance [72]. Therefore, the treatment combination is preferred in almost all Southeast Asian countries (e.g., Thailand, Singapore, Malaysia, and Bhutan). Concordance as a low CAM resistance country, several studies in Thailand and Malaysia showed to have cure rates ≥90% with CAM-based triple therapy [73,74,75,76]. We also should consider the optimal duration of regimens for *H. pylori* eradication. A Cochrane database review reported prolonging treatment duration from 7 to 10 or from 10 to 14 days for AMX-based triple therapy increases *H. pylori* eradication rates, and the optimal duration of therapy for AMX- and MNZ-based triple therapy is at least 14 days [77].

Bismuth quadruple therapy (BQT) is not completely novel but rather represents an enhanced evolution of the older regimen comprising a bismuth salt, TCN, and MNZ [19]. The cure rate of this regimen achieved >90% in the presence of CAM resistance and >85% in regions with a high rate of MNZ resistance [78]. Second Asian Pacific consensus and global guidelines for developing countries recommended BQT as an alternative first-line treatment or as second-line treatment. As second-line treatment, BQT combined with high-dose MNZ (2000 mg/d) resulted in 90.8% by PP efficacy rates in a Taiwanese study [79]. Additionally, consistent with results from Western countries, in regions where MNZ resistance is greater than 40%, the efficacy of BQT in China were more effective with increasing dose and duration therapy [80]. A study in Shanghai, China showed that the efficacy rates of 93.1% (95% CI, 88.1%–98.0%) by PP and 87.9% [95% CI, 81.7%–94.0%) by ITT were obtained using standard-dose MNZ (1600 mg/d) within two weeks [81]. A contrary result was reported in another study which used a low dose of MNZ (800 mg) (64.1% by PP and 59.5% by ITT) [82]. Unfortunately, poor efficacy cure rates were found in several countries in West and South Asia. Most of studies with BQT regimens in Iran, with varying durations (seven-, ten-, and fourteen-days), none had acceptable cure rates [32]. Similar results were also found in Turkey and India [32,83]. Although until now there is no agreement with the duration of BQT, ten or fourteen days are often used durations in these regimens [84]. Lee *et al*., found two weeks of BQT showed more effective than one week treatment (82.6% *vs.* 64.3% by ITT, respectively) [85]. Interestingly, retreatment with BQT was also acceptable as a third-line option (75.0% by PP and 66.7% by ITT) after second-line eradication failures with the same regimen in Korea [86]. Although BQT has been officially substituted for standard triple therapy in high CAM resistance regions, due to its side effects of bismuth is no longer available in many countries, including Japan, Malaysia and Indonesia. Therefore, sequential treatment or a non-BQT (concomitant) treatment is recommended as the alternative first-line treatment in areas of high CAM resistance [87].

A novel non-BQT combination has also been developed. This was originally developed in an attempt to decrease the duration of treatment for *H. pylori* infection. Yanai *et al*., reported that patients receiving 30 mg lansoprazole twice daily, 750 mg AMX twice daily, 200 mg CAM twice daily and 250 mg MNZ twice daily for a week in Japan showed significantly higher cure rates than triple therapy (98.3% by PP and 94.9% by ITT) [88]. A meta-analysis of 2070 patients including 14 studies from Asia revealed a mean *H. pylori* cure rate of 88% by ITT for non-BQT (PPI + CAM + AMX + nitroimidazole) [89]. Two studies in Japan using PPI + CAM + AMX + MNZ as quadruple therapy reported cure rates of 92.5% and 94.5% by ITT [90,91]. On the other hand, two studies in South Korea reported cure rates of only 81.1% by ITT for the PPI + CAM + AMX + MNZ regimen. The cure rate was increased by using LVX-based non-BQT (87.5%–91.4% by ITT) for 5 and 7 days [92,93]. However, we must remember the importance of LVX-based therapy as a rescue treatment after failure with other regimens.

**Table 2:** Treatment regimens for *H. pylori* eradication in Asia [63,64,65,66].

Guidelines	First-Line Treatment	Second-Line Treatment
Second Asia Pasific Consensus 2009	**Standard PPI-based triple therapy: 7–14 days** • PPI, AMX 1 g, CAM 500 mg twice daily • PPI, MNZ 400 mg, CAM 500 mg twice daily • PPI, AMX 1 g, MNZ 400 mg twice daily **Quadruple therapy: 7–14 days** • PPI twice daily, BIS 240 mg twice daily, MNZ 400 mg, twice daily or three times daily, TCN 500 mg four times daily	**Quadruple therapy: 7–14 days** • PPI twice daily, BIS 240 mg twice daily, MNZ 400 mg, twice daily or three times daily, TCN 500 mg four times daily • LVX-based triple therapy: 10 daysPPI, LVX 250 mg (or 500 mg), AMX 1 g twice daily • Rifabutin-based triple therapy: 7–10 daysPPI, rifabutin 150 mg, AMX 1 g twice daily
Global Guidelines for developing countries	**Triple therapy: 7 days** • PPI + AMX + CAM/Furazolidone, twice daily **Quadruple therapy**(CAM resistance > 20%): 7–10 days • PPI twice daily +BIS + TCN +MNZ all four times daily **Quadruple therapy**(no known CAMresistance, BIS unavailable): 14 days • PPI + CAM + MNZ +AMX **Sequential therapy: 10 days** • PPI + AMX for 5 days followed by PPI + CAM and a nitroimidazole (tinidazole) for 5 day	**Quadruple therapy** • PPI + BIS + TCN + MNZ for 10–14 days • PPI + furazolidone + TCN + BIS for 10 days **Triple therapy** • PPI + furazolidone + LVX for 10 days • PPI + AMX + CAM for 7 days • PPI + AMX + LVX for 10 days
Japan 2013	**Triple therapy: 7 days** • AMX 750 mg, CAM 200mg (or 400 mg), and PPI twice daily	**Triple therapy: 7 days** • AMX 750 mg, MNZ 250mg, and PPI twice daily
China 2013	**Triple therapy: 7–14 days** • AMX 1 g (or MNZ 400 mg),CAM 500 mg, and PPI twice daily	**Quadruple therapy: 10–14 days** • BIS 220 mg, TCN 750 mg,MNZ 400 mg twice, and PPI twice daily for 10 or 14 days
Korea 2013	**Triple therapy: 7–14 days** • AMX 1 g, CAM 500 mg, and PPI twice daily	**Quadruple therapy: 7–14 days** • BIS 120 mg four times, TCN 500mg four times, MNZ 500 mg thrice, PPI twice daily for 7–14 days

Abbreviations: CAM: clarithomycin, MNZ: metronidazole, LVX: levofloxacin, AMX: amoxicillin, CIP: ciprofloxacin, TCN: tetracycline.

Although it consists of two dosing periods, sequential therapy is a quadruple therapy consisting of one PPI and three antibiotics. Hypothetically, AMX during the first 5 days of therapy would weaken the bacterial cell wall, which prevents the formation of the channels that prevent CAM from entering the bacterium and hence confer resistance to the antibiotic. Then, in the second phase of therapy, CAM and a nitroimidazole are added for further 5 days. PPI is continued throughout the treatment. Global guidelines recommend sequential therapy as an alternative first-line treatment. A meta-analysis of 46 randomized controlled trials, including several countries in Asia (nine in China, seven in South Korea, and three in Taiwan) found that sequential therapy was superior to 7 days of triple therapy, and marginally superior to 10 days of triple therapy, but not superior to 14 days of triple therapy, BQT, or non-BQT [94]. Another meta-analysis which consisted of nine studies provided data on 3,074 adult patients and found the cure rate was 81.3% by ITT (95%CI: 76.5–85.3) for the sequential therapy group and 70.8% (95%CI: 64.6–76.4) for the triple therapy group [95]. A study in Taiwan conducted a multicenter randomized controlled trial to compare the efficacy of sequential therapy for 10 and 14 days (lansoprazole 30 mg and AMX 1 g for the first 7 days, followed by lansoprazole 30 mg, CAM 500 mg, and MNZ 500 mg for another 7 days; with all drugs given twice daily) with that of 14 days of triple therapy (lansoprazole 30 mg, AMX 1 g, and CAM 500 mg for 14 days; with all drugs given twice daily) as a first-line treatment [96]. They found that the successful cure rate was significantly higher following 14 and 10 days of sequential therapy than 14 days of triple therapy (90.7%, 87.0% and 82.3% by ITT, respectively). In addition, the cure rate of sequential therapy for 10 and 14 days was also affected by resistance to both CAM and MNZ. However there was no significant different cure rates between 10 days sequential therapy and triple therapy in China (72.1% *vs.* 66.4%) [97]. They also found patients in the sequential therapy group with dual CAM and MNZ resistance had a lower cure rate (43.9%) than those with isolated CAM resistance (88.9%, *p* = 0.024) or isolated MNZ resistance (87.8%, *p* < 0.001). A meta-analysis of six studies in South Korea also found sequential therapy did not achieve a sufficient cure rate (79.7% by ITT) [98]. Similar result was also found in India and Iran (76.0% and 76.7% by ITT, respectively) [99,100]. These findings suggest that sequential therapy may be suitable in regions with high prevalence of isolated CAM resistance, but it is unsatisfactory when both CAM and MNZ resistance are present (Table 3). These results also could explain several inconsistent resistance antibiotics data among same country in Table 1. Still considering the geographical differences; China, South Korea, Iran and India may be the high CAM and MNZ resistance countries.

Dual-concomitant (hybrid) therapy is a novel regimen which seems to be effective in areas with dual resistance to MNZ and CAM. This regimen consisting of dual therapy with a PPI and AMX over the first 7 days, followed by a concomitant quadruple therapy containing a PPI plus AMX, CAM, and MNZ over the second 7 days. A Taiwan study reported the cure rate of 99.1% (95%CI, 97.3%–100.0%) by PP analysis and 97.4% by ITT analysis [101]. Moreover, when sequential regimen does not seem to be an appropriate therapy for *H. pylori* eradication in the Iranian population, hybrid therapy showed to be more effective (92.9% by PP and 89.5% by ITT) [100]. The hybrid therapy achieved somewhat better cure rate than the sequential therapy (81.1% *vs.* 79.8% by ITT) in South Korea, but the difference was not statistically significantly [102].

**Table 3:** Resistance region and possibility regimens for *H. pylori* eradication in Asia.

Resistance Region Type	Country	First and Second Line Therapy						Rescue Therapy	
		CAM-Based Triple Therapy	MNZ-Based Triple Therapy	BIS-Based Quadruple Therapy	Non-BIS Quadruple “Concomitant” Therapy	Sequential Therapy	Hybrid Therapy	LVX-Based Triple Therapy	RIF-Based Triple Therapy
Low four antibiotics resistance	Taiwan, Thailand, Malaysia	√	√	√	√	√	√	√	√
High CAM resistance (>20%)	Japan		√	√	√	√	√	√	√
High MNZ resistance (>40%)	China-Hong Kong, Saudi Arabia, Singapore, Bhutan	√		√	√	√	√	√	√
High CAM and MNZ resistance	Turkey, Bahrain, Vietnam			√	√		√	√	√
High CAM and LVX resistance	South Korea		√	√	√	√	√	√	√
High CAM, MNZ and LVX resistance	China-Beijing and Shoutheast China			√	√			√	√
High CAM, MNZ and AMOX resistance	Indonesia			√			√	√	√
High CAM, MNZ, AMOX and LVX (CIP) resistance	Iran, India, Pakistan			√					√

Abbreviations: CAM: clarithromycin, MNZ: metronidazole, LVX: levofloxacin, AMX: amoxicillin, CIP: ciprofloxacin, TCN: tetracycline.

In Iran 207 patients received 10-day hybrid regimen (pantoprazole 40 mg, and AMX 1 g, both twice daily for 10 days; plus CAM 500 mg, and tinidazole, 500 mg, both twice daily just during the last 5 days) and 14-day hybrid regimen (pantoprazole, 40 mg, and AMX, 1 g, both twice a day for 14 days; plus CAM 500 mg, and tinidazole 500 mg, both twice daily just for the last 7 days). They reported that only 14-day hybrid regimen seemed to be an acceptable option for *H. pylori* eradication in Iran [103].

Ideally the best choice of antibiotic regimen should be individualized based on the culture and susceptibility testing using biological material (e.g., urine, sputum) obtained from each patient. However its take time and cost consuming and is not always feasible in *H. pylori*-infected patients because it requires an invasive procedure. Therefore, geographic patterns of antibiotic resistance must be considered. Knowledge of resistance patterns obtained from local or regional antimicrobial surveillance programs and/or local clinical experience are very important for expert decisions to choose the highest predicted success rate regimen. As a general rule, clinicians should prescribe therapeutic regimens that have a >90% or preferably >95% cure rate locally [104]. If no available regimen can achieve >90% eradication, clinicians should use the most effective regimens available locally.

Recently, a novel fully-automated rapid genetic analyzer was developed which was capable of determining CAM resistance (e.g., 23S rRNA gene point mutations of A2143G and A2144G) within 60–120 min, whereas culture tests required 7–10 days [105]. Genotypic resistance test was more convenient and rapid than standard culture susceptibility test, showing promising eradication results with a possibility to determine resistance even from stool samples in Taiwan [106]. In the near future, the follow up of failure eradication with genotypic resistance-guided could evolve to perform tailored therapy even for treatment of naïve patients.

## 4. Efficacy of PPI and Association with CYP2C19 Polymorphisms

The PPI component is important in the eradication regimen related with anti-secretory effects. Alkaline conditions will allow *H. pylori* to grow [107], but it make the acid-labile antibiotics more stable and increases the sensitivity of *H. pylori* to antibiotics [108,109,110]. Furthermore, reducing degradation and increasing stability of acid-sensitive antibacterial agents such as CAM and AMX in the stomach finally produce high concentrations of antibiotics in the gastric juice [111]. Interestingly, PPI also possess modest antimicrobial therapy [112].

Besides omeprazole, since 1995 several new proton-pump inhibitors which also substituted benzimidazole compounds, including lansoprazole, pantoprazole, rabeprazole and esomeprazole have become commercially available. A study in Hong Kong found the efficacy of 7-day rabeprazole- and omeprazole-based triple therapy was similar for the cure of *H. pylori* infection (88% *vs.* 82% by ITT) [113]. On the other hand, the cure rate of rabeprazole-based triple therapy was significantly higher than rabeprazole-based triple therapy in Japan [114]. Moreover, high dose of rabeprazole-based triple therapy (20 mg twice daily) is more effective than its low dose (10 mg twice daily) or omeprazole (20 mg twice daily) in Thailand [115]. A meta-analysis of 35 studies consisted of 5998 patients comparing rabeprazole or esomeprazole with first-generation PPIs (omeprazole, lansoprazole, pantoprazole) [116]. There were no statistically significant differences between esomeprazole and rabeprazole; however both of them showed higher cure rates than for first-generation PPIs (82.3% *vs.* 77.6% and 80.5% *vs.* 76.2%, respectively). The next sub-analysis of PPI dosage were found that only esomeprazole 40 mg, but not esomeprazole 20 mg or any dose of rabeprazole twice daily improved results compared to the first generation regimen (83.5% *vs.* 72.4%). Interesting result was found in a study which reported the extension from 1 to 2 week of standard triple therapy was beneficial for eradication treatment efficacy, especially for patients with a high intragastric bacterial load [117]. A meta-analysis comparing different durations (7, 10 or 14 days) of PPI-based triple therapy consisted of 21 random control trial found the an extended period from one to two weeks influence the efficacy become higher, however it will increased side effects around 5% [118].

The PPIs are inactive in their native form and are rapidly metabolized by the liver. PPI is an acid-activated prodrug. Unfortunately, elimination of PPI occurs very rapidly [119], therefore the efficacy of PPIs is important to keep the PPI plasma level high and influence the gastric acid secretes. Metabolism of PPIs and their pharmacokinetics depend on the cytochrome P450 system, especially CYP2C19 and CYP3A4 polymorphism. Most of PPIs, including omeprazole, lansoprazole and pantoprazole are extensively metabolized by CYP2C19 and CYP3A4 [120]. This influence including pharmacokinetics (peak plasma concentration (Cmax) and area under the curve (AUC) of the plasma concentration) and pharmacodynamics (*i.e*., intragastric pH) of PPI [105]. Only rabeprazole is metabolized to thioether-rabeprazole mainly via a non-enzymatic pathway, with minor involvement of CYP2C19 (approximately only half) [121]. Esomeprazole is a pure *S*-isomer of omeprazole which is less sensitive and in minimal first pass metabolism, undergoes less hydroxylation via CYP2C19 [119]. Regarding the metabolic rate, individuals are classified as extensive metabolizers (EMs) and poor metabolizers (PMs). Genotyping analysis on CYP2C19 found EMs can be divided into homozygous and heterozygous. The homozygous EM genotype which contains non-mutated alleles, wild-type (wt/wt), produces an abundance of the enzyme and metabolizes the PPI at the highest rate. The heterozygous EM which contains one wild-type allele and one mutant allele (*i.e*., wt/m^1^ or wt/m^2^), results in the compromised production of the enzyme and metabolizes the PPI at moderate rates [122]. In the PM genotype both alleles are mutated, which results in a much slower rate of PPI metabolism. PPI concentration of PMs shows 3- to 10-fold higher AUC than homozygous EMs, while heterozygous EMs exhibit a 2- to 3-fold higher AUC, indicating that approximately 80% of the dose is metabolized by CYP2C19 in homozygous EMs [123].

The frequency of the CYP2C19 polymorphism is highly varied among different ethnic populations [124]. The PM phenotype exhibits a lower frequency in Caucasians (2%–5%) compared to Oriental populations (13%–23%), and 15% and 23% of Japanese and Filipinos were PM, respectively. Interestingly only 2.1% of Saudi Arabians were PM [124]. Contrary with the prevalence of heterozygous EM (45%–55%) of Asians higher than Caucasian (25%–27%), Caucasians (70%) have a higher prevalence of homozygous EMs than Asian (30%–40%) [125]. Furuta *et al*., confirmed that CYP2C19 genotype seems to be one of the important factors associated with cure rates in Japanese population. The cure rates of *H. pylori* upon triple therapy comprising PPI, CAM and AMX were 72.7%, 92.1% and 97.8% in the homozygous EM, heterozygous EM and PM, respectively [126]. Interestingly, only with dual therapy (AMX as the only antibiotic and high doses of omeprazole) showing cure rates of 100% in PM [127]. A meta-analysis including 17 studies in Asian patients found only omeprazole- but not lansoprazole- and rabeprazole-based therapies, showed a significant difference in *H. pylori* cure rates between the homozygous EM and PM genotypes [128]. Moreover there was a significant difference between the homozygous EM and heterozygous EM groups (OR = 2.12), as well as the heterozygous EM and PM groups (OR = 2.24). Because CYP2C19 polymorphisms are an important factor affecting the pharmacokinetics of most PPIs, values of intragastric pH, and *H. pylori* eradication rates, genotyping for CYP2C19 polymorphisms could be recommended, especially in Asian populations characterized by a high prevalence of defective CYP2C19 alleles.

A study was administered that tailored PPI therapy according to CYP2C19 genotype. Patients infected with CAM resistant strain were treated with a higher dose of lansoprazole (RMs 30 mg qid; IMs 15 mg qid; and PMs, 15 mg bid for 2 weeks) and AMX (500 mg qid) than CAM sensitive strain (lansoprazole for RMs 30 mg tid; IMs 15 mg tid; PMs, 15 mg bid; and AMX (500 mg tid) for 2 weeks). Higher eradication rates with ITT 96.0% *vs.* 70.0% were observed for tailored PPI therapy [129] A more simple drug selection and dosing dose study with PPI dose qid revealed the eradication rates were similar among different CYP2C19 genotypes. Patients infected with CAM resistant strain were treated with rabeprazole qid, AMX 500 mg qid, and MNZ 250 mg bid, while those infected by CAM sensitive strain were treated with rabeprazole qid, AMX 500 mg qid, and CAM 200 mg bid. In both groups eradication rates were higher (RM 94.3%, IM 98.3% and PM 100.0%) compared to the standard regimen used in Japan (RM 75.7%, IM 81.7% and PM 87.0%). With a cure rate exceeding 95% regardless of CYP2C19 genotype, this method could decrease the cost for genotyping tests [105]. Selection of PPIs metabolized by the non-enzymatic pathway or with minimal first pass metabolism are the first option. Besides that, we suggest increasing the dose of PPI, especially in Asian regions lacking CYP2C19 genotype data, to prevent eradication failure.

## 5. Conclusions

According to current guidelines, standard triple therapy containing a PPI and two antibiotics, CAM and AMX/MNZ, is still the preferred first-line regimen for treatment of *H. pylori* infection. However, in recent years, the efficacy of legacy triple regimens has been seriously challenged, and their rates of effectiveness have fallen. Moreover, some regions in Asia are exhibiting emerging patterns of antimicrobial resistance. CAM-containing triple therapy should be abandoned, as it is no longer effective unless local CAM resistance is low or culture confirms susceptibility to CAM. More effective CAM-based regimens are now replacing standard triple therapies as empirical first-line treatments, on the basis of local rates of *H. pylori* antimicrobial resistance (Table 3). These regimens include bismuth and non-bismuth quadruple, sequential, and dual-concomitant (hybrid) regimens, although prevention should be the ultimate solution in Asian countries with a high prevalence of the infection and increased resistance to the antibiotics. To date an efficient vaccine has not yet been developed. Local antibiotic resistance screening updates, selection of appropriate first-line regimens, selection of non-enzymatic PPIs and/or increased doses of PPI, detailed evaluation of patients’ prior antibiotic usage are all essential to combat *H. pylori* antibiotic resistance in Asia.
